# 

**DOI:** 10.1017/S0144686X18000855

**Published:** 2018-10

**Authors:** BEATRICE HALE

**Affiliations:** Independent researcher, New Zealand

This book is a valuable addition to the growing number of mobilities studies. Combining memory and mobility in such detail provides exceptionally useful information on the situation of migrants and refugees. Its discussion of the concept of ‘home’ makes it invaluable for a wide readership. Of course, this is important in ongoing work with refugees and migrants but its discussion of ‘home’ has a much wider impact and is relevant to many of us working in aged care. The richness of the ethnographic accounts provides a detailed context of using memories to begin and continue living in new countries, and the focus on the process of building homes elsewhere teaches us more than the means of settling in a new country. I am reminded of settling into residential care.

However, to focus on the book's context, it is also a useful reminder of the value of knowing individual personal history and the process of working through such history, which is so useful for social workers, educationists, medical personnel and, in fact, anyone who is building a relationship with and for migrants and refugees. As other reviewers have said, *Memories on the Move* is a must-read for anthropologists, sociologists, historians and political scientists.

The book is subdivided into three main parts. After the Introduction, which gives us the book outline and a discussion of definitions, the first chapter is ‘Memories on the Move – Experiencing Mobility, Rethinking the Past’; the second section is the ‘Mnemonic Dimensions of Exiles’, looking at memories of war and exile across time and place, war and displacement, commemorating identity in exile, among other things; and the third is ‘Legacies and Politics of Memory’, which incorporates several more interesting chapters.

Now to some details of the book itself.

The editors, Monika Palmberger and Jelena Tošić, are anthropologists from the University of Vienna and they have assembled a striking line-up of expertise of anthropologists whose research and publication histories support their inclusion in this volume.

The Introduction is an interesting discussion of mobilities, requesting that the ‘interplay of movement and memory calls for closer inspection’. As the editors say,

The different forms of lived and imagined (im)mobility with which the present volume engages include refugees remembering and ‘recreating’ in their settlements or during return journeys to homes from which they were expelled. We show in this volume that remembering – as well as forgetting or even ‘amnesia’ – is actually a constitutive part of movement. (p. 2)

These words represent the in-depth context of the people, and to quote a Maori saying, ‘It is people, it is people, it is people’. We can see that the focus on individuals, their memories, mobilities and the process of recreation provides a richness of knowledge which is invaluable to many disciplines, and not only anthropology. However, one of anthropology's tasks, I believe, is to make visible how people live and feel. This book brings home to us how refugees and migrants live and feel. It is at times an incredibly hurtful book, highly sensitive with its data, and its focus on and ensuring the visibility of the enforced changing of ‘home’ and the re-establishment of home.

The chapter on ‘A Past that Hurts’ (Chapter 10) is enough to show us that, as is the chapter on ‘Long-distance Nationalism and the Politics of Memory’. Along with other chapters, the editors give us a rich and complex view of issues of mobility and remembering. Also Karen F. Olwig's chapter on ‘Moving Memories and Memories of Moving: Some Afterthoughts’, discusses four ethnographic analyses of ethno-religious communities, with their close association between the assertion of a particular past and the establishment and sustaining of a distinct community. She refers to the transnational community of Kurdish people in Iraqi Kurdistan and abroad, the Jewish-Polish disaspora, Palestinian refugees on the West Bank and Bangladeshi immigrants in Portugal. She quotes Natalie Alonso Rey's study of memory work (Chapter 5) where some women display family photographs in their private homes, but others find the pain of being absent from the homeland so strong that they keep family photographs tucked away out of sight.

And there's more.

Eastmond talks of an ‘elderly couple’ in their sixties, feeling little motivation to settle in Sweden, and they found the idea of dying far from home a painful one. They spent most days with their family, talking about the past, and seemed to make sense of the new environment by comparing it – foodstuffs, prices and so on – to Bosnia; and they got themselves a garden and kept busy with flowers and vegetables. As Eastmond analyses it, they seek a sense of continuity by ‘wrapping a cloak of familiarity around a new landscape; imagining a new surrounding through the memory of their homeland’ (p. 28). Eastmond concludes that this couple remained faithful to Tito's Yugoslavia, and its political project, and were silent about ethnic violence. The chapter is well worth reading for her analysis of memory and mobility:

In exile, they engaged their memories of life at home before the war … their original home in Bosnia where they had spent most of their lives, became an idealized construct that helped them adjust to life in a new context. (p. 39)

This complex detail of memory and mobility is vivid, and sometimes it raises further questions. The exploration of the process by people enforced on the move remembering, and communicating and working with their memories to create a sense of belonging elsewhere from their exilic homes is a fascinating, unputdown-able read.

And its relevance for this journal is acute. How do older people feel about moving from home to care? What are the intermediate stages, of say, treatment and assessment, before care is offered and accepted? The book in its methodology and its findings has much to offer us in aged care studies, as well as its insightful analyses of recreating home by refugees and migrants.

I can obviously strongly recommend this book as an invaluable source for a number of disciplines, and certainly invaluable in a consideration of aged care and the movement of older people, even within their own country.

